# Signal enrichment with strain-level resolution in metagenomes using topological data analysis

**DOI:** 10.1186/s12864-019-5490-y

**Published:** 2019-04-04

**Authors:** Aldo Guzmán-Sáenz, Niina Haiminen, Saugata Basu, Laxmi Parida

**Affiliations:** 1 Computational Biology Center, IBM T. J. Watson Research Center, Yorktown Heights, NY USA; 2 Department of Mathematics, Purdue University, West Lafayette, IN USA

**Keywords:** Metagenomics, Topological data analysis, Multi-mapping reads, False positives

## Abstract

**Background:**

A metagenome is a collection of genomes, usually in a micro-environment, and sequencing a metagenomic sample *en masse* is a powerful means for investigating the community of the constituent microorganisms. One of the challenges is in distinguishing between similar organisms due to rampant multiple possible assignments of sequencing reads, resulting in false positive identifications. We map the problem to a topological data analysis (TDA) framework that extracts information from the geometric structure of data. Here the structure is defined by multi-way relationships between the sequencing reads using a reference database.

**Results:**

Based primarily on the patterns of co-mapping of the reads to multiple organisms in the reference database, we use two models: one a subcomplex of a Barycentric subdivision complex and the other a Čech complex. The Barycentric subcomplex allows a natural mapping of the reads along with their coverage of organisms while the Čech complex takes simply the number of reads into account to map the problem to homology computation. Using simulated genome mixtures we show not just enrichment of signal but also microbe identification with strain-level resolution.

**Conclusions:**

In particular, in the most refractory of cases where alternative algorithms that exploit unique reads (i.e., mapped to unique organisms) fail, we show that the TDA approach continues to show consistent performance. The Čech model that uses less information is equally effective, suggesting that even partial information when augmented with the appropriate structure is quite powerful.

## Background

A metagenome is a collection of genomes in a micro-environment, such as the gut of an animal, bottom of an ocean, or soil. This captures the influence of the immediate environment on the phenotype of an organism. For instance, one of the factors in the safety of our food supply chain is knowing the microbiome in the food [1]. The state of disease and health of a host has been shown to be related to the microbiomes in its gut [2]. The sturdiness or weakness of a plant is shown to be related to its soil microbiome [3]. It is turning out that these microorganisms are perhaps playing a much bigger role than earlier anticipated. The DNA technology to capture these organisms also has been disruptive in the area of microbiology, i.e., each organism does not need to be cultured individually before sequencing but the entire volume of samples can be put through the sequencing process en masse [4]. The obvious advantage is that the recalcitrant organisms that were resistant to being cultured no longer pose a problem, as long as careful sample processing is performed to avoid sequencing biases [5]. However, there are two major challenges with the sequencing approach. Firstly, the completeness and correctness of reference databases limits the power of detection. The database of reference sequences must systematically be updated when new genomes become available. Secondly, no matter how complete and accurate the databases are, there is the problem of correctly assigning sequencing reads when distinct organisms with very similar genomes are present. In this paper, we address the second challenge of accurately detecting the organisms present in a sample, given a database.

Characteristics of the short sequencing reads frequently results in them being mapped to multiple reference genomes in the database, even under very strict matching criteria. Thus when the database organisms are very similar to each other there is usually a substantial dearth of reads assigned to unique organisms. As a consequence, most solution pipelines yield mapping results that are riddled with false positives. In fact, in (microbial) simulation studies we find that often a large percentage of the predicted potential organisms, using standard pipelines from literature, are false positives. A recent benchmark study found the number of species reported for the same sample varying by orders of magnitude, depending on the classifier used [6]. Popular methods for metagenomic read classification include Kraken [7], CLARK [8], MetaPhlAn [9] and others.

Topological data analysis (TDA) is emerging as a promising approach for analyzing large genomic datasets for a variety of questions [10–12], with already demonstrated applications in evolutionary biology, cancer genomics, and analysis of complex diseases. TDA extracts information from the geometric structure of data; in our application the structure is defined by the relationships between sequencing reads and organisms in a reference database.

Based primarily on the patterns of co-mapping of reads to the organisms in a reference database, we use a subcomplex of a Barycentric subdivision complex to model the multi-way maps of reads, along with the extent of read coverage on the respective organisms. This subcomplex allows a natural mapping of our problem to homology computation and interpretation. We test this approach on the special scenario (dearth of uniquely mapped reads) and find that using an appropriate voting function on the typical bar diagrams of TDA, we can sort the organisms to separate true positives from false positives. Next, we test if a reduced information set, that is, only the number of reads without utilizing their lengths or coverage when combined with TDA is powerful enough to enrich for true positives. We observe success in this scenario as well, suggesting that the use of topology captures the non-obvious structure defined by the reads promiscuously mapping to multiple organisms. All the data used in the paper are simulated reads by necessity since it is the most reliable way of knowing the ground truth, to assess the results.

## Methods

In this section we map the problem to topological data analysis, more specifically to bar diagrams arising from persistent homology.

### Motivation and problem statement

Given a reference database and a collection of metagenomic reads from the sequencing process, we denote by *Z* the set of all possible organisms, resulting from a reads-to-organism-mapping (ROM) pipeline: *Z*={*X*_1_,*X*_2_,...,*X*_*N*_}. We treat all the reads of the pipeline output equally, i.e., do not consider the extent of the match. The rationale is that these characteristics have already been used by the pipeline to produce the output. Hence we focus on the subsequent information gathered from the pipeline: the relationship between the reads an the organisms.

Consider the bipartite graph of reads and organisms, where an edge connects a read to the assigned organism, that captures the ROM relationships completely. But a much more natural fit is to a simplicial complex where a *k*-simplex, *k*>0 (analogous to edge in a graph) is a read and the 0-simplex (analogous to a vertex in a graph) is an organism. Furthermore each of the the *k*-simplices are weighted, i.e., the weight is the number of reads that map to exactly these *k* organisms, which is not immediately apparent from the bipartite graph. The next question is whether the topological features of this naturally emerging simplicial complex can be exploited to solve the problem of false positives. In other words, is there some hidden characteristics of the ROM that separate the true from the false organisms.

In this paper, to make it more tractable, we simplify the problem as follows: Given the ROM pipeline output, obtain an order for the elements of *Z* as $X_{i_{1}}, X_{i_{2}},..., X_{i_{N}}$, such that the top of the list is enriched for true positives (TPs). In a practical scenario, a threshold can be used to snip away the bottom of the list to eliminate potential false positive candidates. Further, the retained true positives can be used to rescue and re-assign the reads that had been misassigned due to the false positives.

### Model I: Barycentric subdivision

Each organism *X*_*j*_ has a collection of reads mapped to it. For each subset $Y = \{X_{1},\ldots,X_{k}\} \in 2^{\mathcal {X}}$, we denote by *S*_*Y*_ the set of reads mapped to the organisms *X*_1_,*X*_2_,...,*X*_*k*_, and none other.

For instance, this implies that $ S_{\{X_{1},X_{2}\}} \cap S_{\{X_{1},X_{2},X_{3}\}} = \emptyset $. In general,

$$S_{Y} \cap S_{Y^{\prime}} = \emptyset, \text{whenever} Y \neq Y^{\prime}.$$

#### **Definition 1**

For $Y \in 2^{\mathcal {X}}$, the area *a*_*Y*_ is defined to be the sum total of all the lengths of the reads in *S*_*Y*_.

#### **Definition 2**

For $Y \in 2^{\mathcal {X}}$ and *X*_*j*_∈*Y* we denote the length of the genome of the organism *X*_*j*_ covered by the reads in *S*_*Y*_ by $l_{Y,X_{j}}$, and define

$$d_{Y}(X_{j}) = \frac{1}{m^{2}}\left(\frac{a_{Y} }{l_{Y,X_{j}}}\right),$$

where *m*=|*Y*|.

This is motivated by the fact that if there is true overlap in the *m* organisms, then the depth *d* of coverage of each organism is magnified by some factor of *m*. Note that it is quite possible that for *Y*={*X*_1_,*X*_2_,…,*X*_*m*_}, the values *d*_*Y*_(*X*_1_),*d*_*Y*_(*X*_2_),*d*_*Y*_(*X*_3_), …, *d*_*Y*_(*X*_*m*_) are all distinct.

#### **Example 1**

**Fig. 1:**
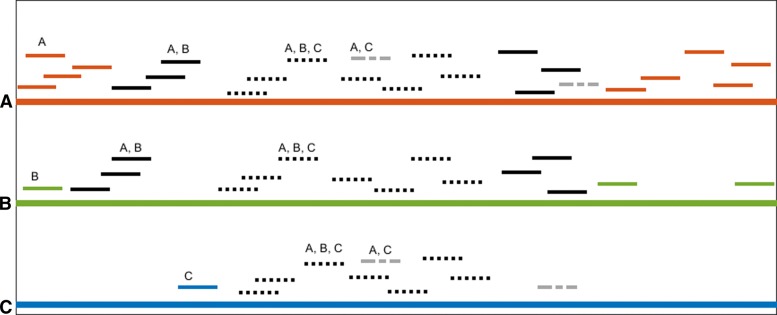
A set of organisms {**a**,**b**,**c**} whose genomes are represented with a red, green, and blue line respectively. Reads mapped to the organisms are shown with shorter lines. The line colors and styles for the reads indicate unique and shared reads, according to the given labeling

**Fig. 2:**
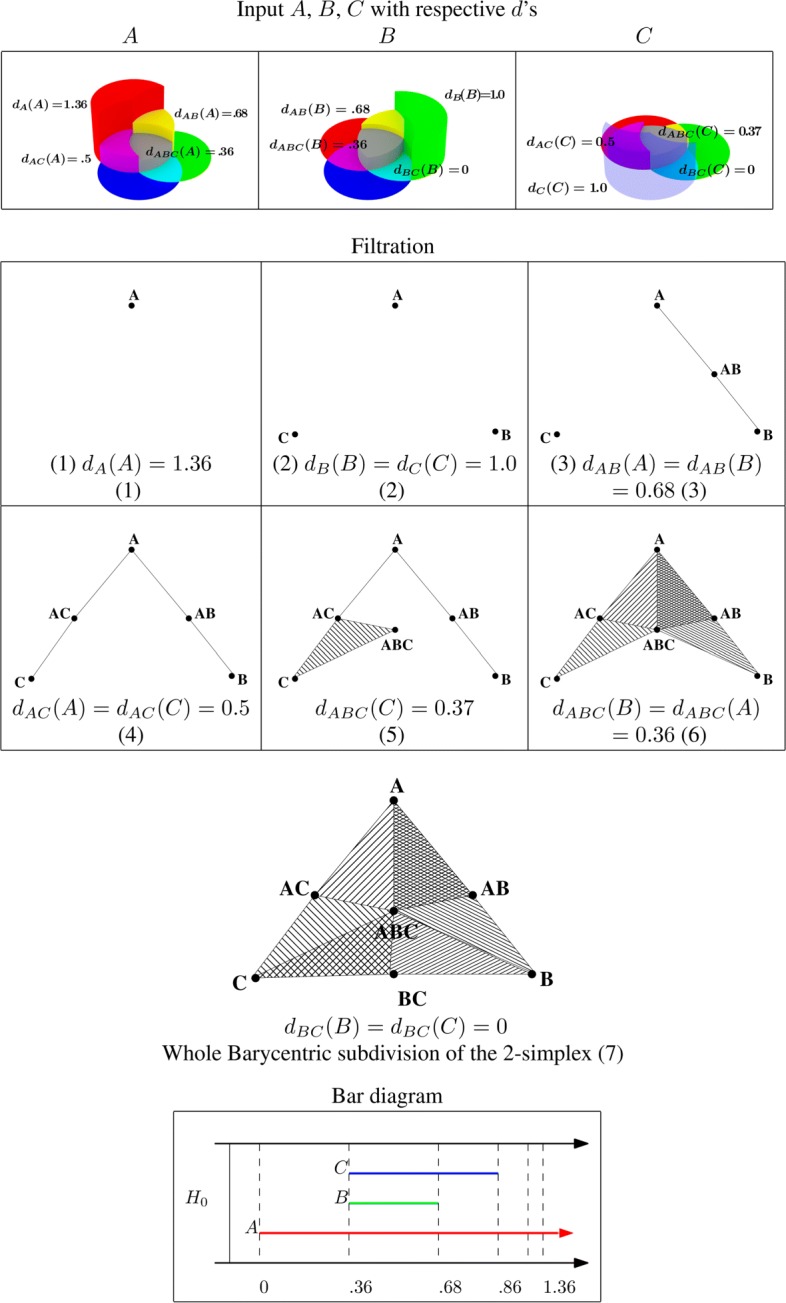
Top: Values of depth *d* for the three organisms and read mapping shown in Fig. 1. Middle: Filtration of the Barycentric subdivision of the 2-simplex spanned by *A*, *B*, *C*. Bottom: The bar diagram in degree zero with organisms associated to bars

Consider a set of 3 organisms $\mathcal {X}=\{A,B,C\}$. A graphical representation of the underlying mapped reads is shown in Fig. 1 and the values of *d*_*Y*_ for each set $Y\in 2^{\mathcal {X}}$ are given in Fig. 2 (top).

The depth values were computed after simulating a small set of reads’ placement on three reference genomes. For example, in this case no reads match both *B* and *C* (and not *A*), hence *d*_*BC*_(*B*)=*d*_*BC*_(*C*)=0. However, some reads match all three organisms, hence *d*_*ABC*_>0. The values *d*_*A*_(*A*),*d*_*B*_(*B*),*d*_*C*_(*C*) indicate the depths of *A, B, C* based on their uniquely mapping reads.

#### **Definition 3**

**(set*Y***^***t***^** for a*t*≥0)**For $Y \in 2^{\mathcal {X}}$ and $t \in \mathbb {R}, t \geq 0$, we denote

$$Y^{t} = \{ X_{j} \in Y \mid d_{Y}(X_{j}) \geq t\}.$$

Note that the sets (*X*_*i*_)_1≤*i*≤*N*_ give a *cover* of the set of reads, while the sets $(S_{Y})_{Y \in 2^{\mathcal {X}}}$ form a *partition* of the reads. This corresponds to the collection of organisms present at a time *t*, mapped to some stage of a filtration of a simplicial complex (see Definition 5).

#### **Definition 4**

Given any finite set *S*, a *simplicial complex* with vertices in *S*, is a collection *K* of subsets of *S*, with the property that if *A*∈*K*, then for all *B*∈2^*S*^ with *B*⊂*A*, *B*∈*K*. A subcollection *L* of *K* is a subcomplex of *K* if *L* is a simplicial complex.

In other words, *K* is closed under the operation of “taking subsets". This last property is crucial for the definition of the simplicial homology groups, *H*_∗_(*K*), associated to a simplicial complex *K*.

#### **Definition 5**

A finite filtered simplicial complex is a finite sequence *K*_0_⊂*K*_1_⊂⋯⊂*K*_*N*_ of simplicial complexes. In other words, *K*_*i*_ is a subcomplex of *K*_*j*_ for *i*≤*j*. A filtration of a simplicial complex *K* is a filtered simplicial complex with *K*_*N*_=*K*.

This particular setup allows us to apply *persistent homology* to our problem.

To any finite simplicial complex *K* one can associate for each *j*≥0, a finite dimensional vector space *H*_*j*_(*K,Q*) (called the *j-th simplicial homology group of K*). Moreover, if we have a filtration of a simplicial complex *K* as above, then each inclusion *i*_*s,t*_:*K*_*s*_→*K*_*t*_,*s*≤*t* induces a *linear map*, *i*_*s,t*,∗_:*H*_*j*_(*K*_*s*_,*Q*)→*H*_*j*_(*K*_*t*_,*Q*). The images of these linear maps are usually called the *persistent homology groups* of the filtration, and their dimensions (i.e. the ranks of the maps *i*_*s,t*,∗_) determine the so called “bar diagram" associated to the filtration. Intuitively, the dimensions of the vector spaces *H*_*j*_(*K,Q*) (also called the *j*-th Betti number of the simplicial complex *K*) measure the number of independent *j*-dimensional cycles which are not boundaries of any (*j*+1)-dimensional subcomplex of *K* (so called *j*-dimensional holes), and the *bar diagram* of the filtration is a record of the “times" of the births and deaths of these “homology classes", where we think of the sequence (*K*_*t*_)_*t*∈[0,*N*]_ as a complex growing with time *t*. Each bar in the bar diagram represents the interval in time in which a homology class *persisted*. We refer the reader to the book by Edelsbrunner and Harer [13] for further details about persistent homology and its use in Topological Data Analysis.

However, notice that for each $t \in \mathbb {R}, t \geq 0$, the set system $(Y^{t})_{Y \in 2^{\mathcal {X}}}$ does not necessarily satisfy the condition of being closed under taking subsets – and hence, does not necessarily form a simplicial complex with $\mathcal {X}$ as the set of vertices.

Instead, we utilize the following construction (a simplicial subcomplex of a *barycentric subdivision*, detailed at the beginning of this section) that does produce a simplicial complex (in fact a filtration of complexes), and which is also naturally aligned with the various functions *d*_*Y*_(·) defined earlier.

Intuitively, the simplices of the barycentric complex correspond to chains of subsets of $\mathcal {X}$. For example, if $X_{1},X_{2},X_{3} \in \mathcal {X}$, then the sequence {*X*_1_}⊂{*X*_1_,*X*_2_}⊂{*X*_1_,*X*_2_,*X*_3_} is a chain in the partially ordered set $2^{\mathcal {X}}$ (ordered by inclusion). Given *t*≥0, we include the chain {*X*_1_}⊂{*X*_1_,*X*_2_}⊂{*X*_1_,*X*_2_,*X*_3_} in the barycentric complex iff {*X*_1_}^*t*^∩{*X*_1_,*X*_2_}^*t*^∩{*X*_1_,*X*_2_,*X*_3_}^*t*^≠*∅*.

#### Barycentric subdivision and its subcomplex of interest

We now define more precisely the barycentric complex, and the subcomplex we will use.

Let $\Delta _{\mathcal {X}} $ denote the $(|\mathcal {X}|-1)$-dimensional simplex with vertex set $\mathcal {X}$.

**Fig. 3:**
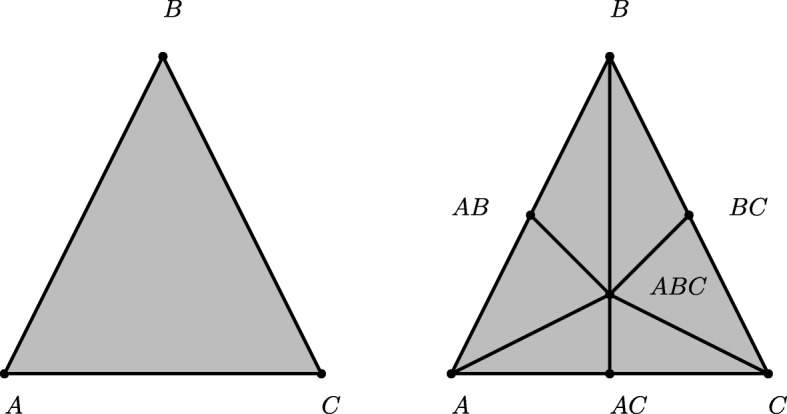
Left: The 2-simplex *Δ*=2^{*A,B,C*}^. Right: Its barycentric subdivision *Δ*^′^

A sequence $\boldsymbol {\sigma } = (Y_{0},\ldots,Y_{p}), Y_{i} \in 2^{\mathcal {X}}$ is called a *chain* of the poset $2^{\mathcal {X}}$ (ordered by inclusion) if $Y_{0} \subsetneq Y_{1} \subsetneq \cdots \subsetneq Y_{p}$. The first barycentric subdivision of $\Delta ^{\prime }_{\mathcal {X}}$ of $\Delta _{\mathcal {X}}$ (see Fig. 3) is then defined by

1 $$  \Delta^{\prime}_{\mathcal{X}} = \left\{\mathbf{Y} = (Y_{0},\ldots,Y_{p}) \mid \mathbf{Y} \text{is a chain in}\ 2^{\mathcal{X}}\right\}.  $$

Now let $\mathcal {X}$ be a finite set, and let $\mathbf {d} = (d_{Y}:Y \rightarrow \mathbb {R})_{Y \in 2^{\mathcal {X}}}$ be a tuple of maps. For each $t \in \mathbb {R}$, we denote by $\Delta ^{\prime }_{\mathcal {X},\mathbf {d}}(t)$ the subcomplex of $\Delta _{\mathcal {X}}^{\prime }$ defined as follows.

For $t \in \mathbb {R}$, and $\mathbf {Y} = (Y_{0},\ldots,Y_{p}) \in \Delta ^{\prime }_{\mathcal {X}}$, let

2 $$  \mathbf{Y}_{\mathbf{d}}(t) =\bigcap_{0 \leq i \leq p} Y_{i}^{t} =\bigcap_{0 \leq i \leq p} \{X \in Y_{i} \mid d_{Y_{i}}(X) \geq t \},  $$

and set

3 $$  \Delta^{\prime}_{\mathcal{X},\mathbf{d}}(t) = \left\{\mathbf{Y} = (Y_{0},\ldots,Y_{p}) \in \Delta^{\prime}_{\mathcal{X}} \mid \mathbf{Y}_{\mathbf{d}}(t) \neq \emptyset\right\}.  $$

We continue with Example 1 of three organisms *A,B,C*. In this case, the vertices of $\Delta ^{\prime }_{\mathcal {X}}$ are {*A,B,C,AB,AC,BC,ABC*}, where we write *A* for {*A*}, *AB* for {*A,B*} and so on. The top part of Fig. 2 implies that we have only 7 different values for our functions *d*_*Y*_. So we only need to specify the following collection of such $\Delta ^{\prime }_{\mathcal {X},\mathbf {d}}(t)$ (taking into account Remark 2 below):

$$ {\begin{aligned} \Delta^{\prime}_{\mathcal{X},\mathbf{d}}(0) &= \{[A]\}\\ \Delta^{\prime}_{\mathcal{X},\mathbf{d}}(.36) &= \Delta^{\prime}_{\mathcal{X},\mathbf{d}}(0)\cup\{[B],[C]\} \\ \Delta^{\prime}_{\mathcal{X},\mathbf{d}}(.68) &=\Delta^{\prime}_{\mathcal{X},\mathbf{d}}(.36)\cup \{[AB],[A,AB],[B,AB]\}\\ \Delta^{\prime}_{\mathcal{X},\mathbf{d}}(.86) &= \Delta^{\prime}_{\mathcal{X},\mathbf{d}}(.68)\cup\{[AC],[A,AC],[C,AC]\} \\ \Delta^{\prime}_{\mathcal{X},\mathbf{d}}(.99) &= \Delta^{\prime}_{\mathcal{X},\mathbf{d}}(.86)\cup\{[ABC],[C,ABC],[AC,ABC],[C,AC,ABC]\}\\ \Delta^{\prime}_{\mathcal{X},\mathbf{d}}(1) &=\Delta^{\prime}_{\mathcal{X},\mathbf{d}}(.99)\cup \{[A,ABC],[B,ABC],[AB,ABC],[A,AB,ABC],\\ & \hspace{3cm} [A,AC,ABC],[B,AB,ABC]\} \\ \Delta^{\prime}_{\mathcal{X},\mathbf{d}}(1.36) &=\Delta^{\prime}_{\mathcal{X}}. \end{aligned}}  $$

See Fig. 2 (middle) for a graphic representation of this filtered simplicial complex.

##### **Fact 1**

$\Delta ^{\prime }_{\mathcal {X},\mathbf {d}}(t)$is a simplicial complex with vertices in $2^{\mathcal {X}}$. Moreover, for any sequence *t*_0_>*t*_1_>…>*t*_*N*_,

$$\Delta^{\prime}_{\mathcal{X},\mathbf{d}}(t_{0}) \subset \Delta^{\prime}_{\mathcal{X},\mathbf{d}}(t_{1}) \subset \cdots \subset \Delta^{\prime}_{\mathcal{X},\mathbf{d}}(t_{N}).$$

##### *Proof*

If $\mathbf {Y} = (Y_{0},\ldots,Y_{p})\in \Delta ^{\prime }_{\mathcal {X},\mathbf {d}}(t)$ is a chain, and $\mathbf {Z} = (Y_{i_{0}},\ldots,Y_{i_{q}}), 0 \leq i_{0} < \cdots < i_{q} \leq p$, is a subchain, then it is straightforward to check that

$$\mathbf{Y}_{\mathbf{d}}(t) \subset \mathbf{Z}_{\mathbf{d}}(t). $$

Since **Y**_**d**_(*t*) is not empty, **Z**_**d**_(*t*) is not empty as well, so $ \mathbf {Z}\in \Delta ^{\prime }_{\mathcal {X},\mathbf {d}}(t)$.

Now assume that *t*_*i*_>*t*_*j*_ for some *i,j*∈{0,…,*N*}, and let $\mathbf {Y}\in \Delta ^{\prime }_{\mathcal {X},\mathbf {d}}(t_{i})$. We have that if **Y**_**d**_(*t*_*i*_)≠*∅* then there exists some *X* such that for all *Y*_*i*_ in **Y** we have $d_{Y_{i}}(X)\geq t_{i}> t_{j}$, therefore $Y\in \Delta ^{\prime }_{\mathcal {X},\mathbf {d}}(t_{j})$. □

#### Filtration

Armed with Fact 1 above, we have the following definition.

##### **Definition 6**

Define the filtered simplicial complex

4 $$ \Delta^{\prime}_{\mathcal{X},\mathbf{d}} = \bigcup_{t\in\mathbb{R}} \Delta^{\prime}_{\mathcal{X},\mathbf{d}}(t).  $$

This definition means that each of the simplicial complexes $\Delta ^{\prime }_{\mathcal {X},\mathbf {d}}(t)$ can be thought as a particular point in time of the filtration $\Delta ^{\prime }_{\mathcal {X},\mathbf {d}}$.

While the constructions so far deal with theoretical considerations, for actual computations we use the following remarks, which were in fact used to compute the filtration values for Example 1.

##### **Remark 1**

Let $\boldsymbol {\sigma } = (Y_{0},\ldots,Y_{p}) \in \Delta ^{\prime }_{\mathcal {X}}$, and let

$$\tau = \bigcap_{Y\in\boldsymbol{\sigma}} Y. $$

Define *t*_0_ by

5 $$  t_{0}= \max_{X\in \tau}\min_{Y\in \sigma} \lbrace d_{Y}(X)\rbrace.  $$

Then *t*_0_ is the time of addition of ***σ*** to $\Delta ^{\prime }_{\mathcal {X},\mathbf {d}}$.

##### **Remark 2**

To make $ \Delta ^{\prime }_{\mathcal {X},\mathbf {d}} $ covariant with respect to *t*, we use the change of variable *t*^′^=*m*−*t* where *m* denotes the maximum value attained by the functions *d*_*Y*_.

From now on, unless stated otherwise, we will use the aforementioned change of variable.

**Voting Scheme.** Next we develop a voting scheme for all organisms that aims to solve the problem of ordering the organisms from true to false positives, using tools from algebraic topology. We refer the reader to Subsection 2.6 and Section 3 of [14] for the definition of persistent homology of a filtered simplicial complex, and a characterization in terms of $\mathcal {P}$-intervals respectively. In particular, using Corollary 3.1 of said article we have the following definition.

##### **Definition 7**

Let *K* be a filtered simplicial complex and *i*≥0 an integer. We define the *i*-th degree bar diagram of *K* as the collection of $\mathcal {P}$-intervals associated to the *i*-th degree persistent homology of *K*.

We now consider the persistent homology of the filtered complex $\Delta ^{\prime }_{\mathcal {X},\mathbf {d}}$. For $0\leq i \leq |\mathcal {X}| - 1$ let $\mathcal {B}_{i}$ be a set of generators for the *i*-th degree persistent homology of $\Delta ^{\prime }_{\mathcal {X},\mathbf {d}}$, with fixed representing cycles, and $\mathcal {B} = \bigcup {\mathcal {B}_{i}}$. We can put these generators in a bijection with bars of the bar diagram of $\Delta ^{\prime }_{\mathcal {X},\mathbf {d}}$.

To help motivate the definition of the votings for each organism, we first list some of the components of the final formula. At each degree *i*, we assign to each organism *X* a collection of generators in $ \mathcal {B}_{i}$ such that these generators witness *X* as a contributor to the features of our complex. For instance, for each *X*, consider all *B* such that *X* appears in its representing cycle (remember that such cycle is a linear combination of simplices, containing organisms as vertices). This gives rise to functions $F_{i}:\mathcal {X}\to 2^{\mathcal {B}_{i}}$.

For each generator $B\in \mathcal {B}$, let start(*B*) and end(B) denote its beginning and end as a bar. Consider now a strictly decreasing function $f:\mathbb {R}_{\geq 0} \to \mathbb {R}_{\geq 0}$ with *f*(*x*)→0 as *x*→*∞*; this function will modulate the contributions of each bar to the voting value of a given organism along the filtration value, while a decreasing function $g:\mathbb {R}_{\geq 0} \to \mathbb {R}_{\geq 0}$ will modulate the contributions along homology degree.

With all this in place, the vote *v*(*X*) for organism *X* is computed as follows:

6 $$\begin{array}{@{}rcl@{}} v(X) &= &\sum_{i} g(i) \left(\sum_{B\in F_{i}(X)} \left(f(\text{start}(B)) - f(\text{end}(B)) \right) \right).  \end{array} $$

Examples of *f,g* include $f(t) = \frac {1}{t+1}$ and $g(i) = \frac {1}{i+1}$, which is what we used to obtain the results in this paper.

Continuing our example from Figs. 1and 2 (bottom) shows the resulting bar diagram with associated generators after application of the mentioned procedure on the depth values. In this case there is only non-trivial homology in 0-th degree. After applying the voting scheme described above on the bar diagram shown in Fig. 2, the votes are *v*(*A*)=1.00,*v*(*B*)=0.14,*v*(*C*)=0.20.

### Model II: Čech complex

Recall that *Z*={*X*_1_,*X*_2_,...,*X*_*N*_} is the set of all possible organisms, resulting from an ROM pipeline. Let $ \left (Y = S_{X_{1}X_{2}..X_{k}}\right) \in 2^{Z}$ be the set of reads associated with organisms *X*_1_, *X*_2_,..., and *X*_*k*_. Note that for all *i, j, k*,...,

7 $$ |S_{X_{i}}| \geq |S_{X_{i}X_{j}}| \geq |S_{X_{i}X_{j}X_{k}}| \geq...   $$

The *t* (time) order filtration is *t*_max_ down to *t*_min_ in say steps of 1. A *k*-simplex on *X*_1_,*X*_2_,...,*X*_*k*_,*X*_*k*+1_ at time *t* is introduced if $|S_{X_{1}X_{2}...X_{k}X_{k+1}}| \geq t$. Note that if the *k*-simplex on *X*_1_,*X*_2_,...,*X*_*k*_,*X*_*k*+1_ belongs to the complex, then so does each (*k*−1)-simplex on *X*_1_,*X*_2_,...,*X*_*k*_,*X*_*k*+1_ since, based on Eq. 7, for 1≤*i*≤*k*,

$$|S_{Y_{1}Y_{2}Y_{3}...Y_{k}}| \!\geq\! |S_{X_{1}X_{2}X_{3}...X_{k}X_{k+1}}|, \text{where}\ \!Y_{i} \!\!\in\! \{X_{1}, X_{2}, X_{3}, \!...,\! X_{k}, X_{k+\!1}\}. $$

Each node or organism *X* gets a true-positive score *v*(*X*) as follows: In the bar diagram, let *b* be the *h*-simplex =*X*_0_*X*_1_...*X*_*h*_ with bar length denoted as len (*b*). In this model, the vote *v*(*X*) for organism *X* is computed as follows:

8 $$\begin{array}{@{}rcl@{}} v(X)&= &\sum_{h} \left(\sum_{b \in H_{h}, X \in b} h \times \text{len}(b)\right)  \end{array} $$

**Fig. 4:**
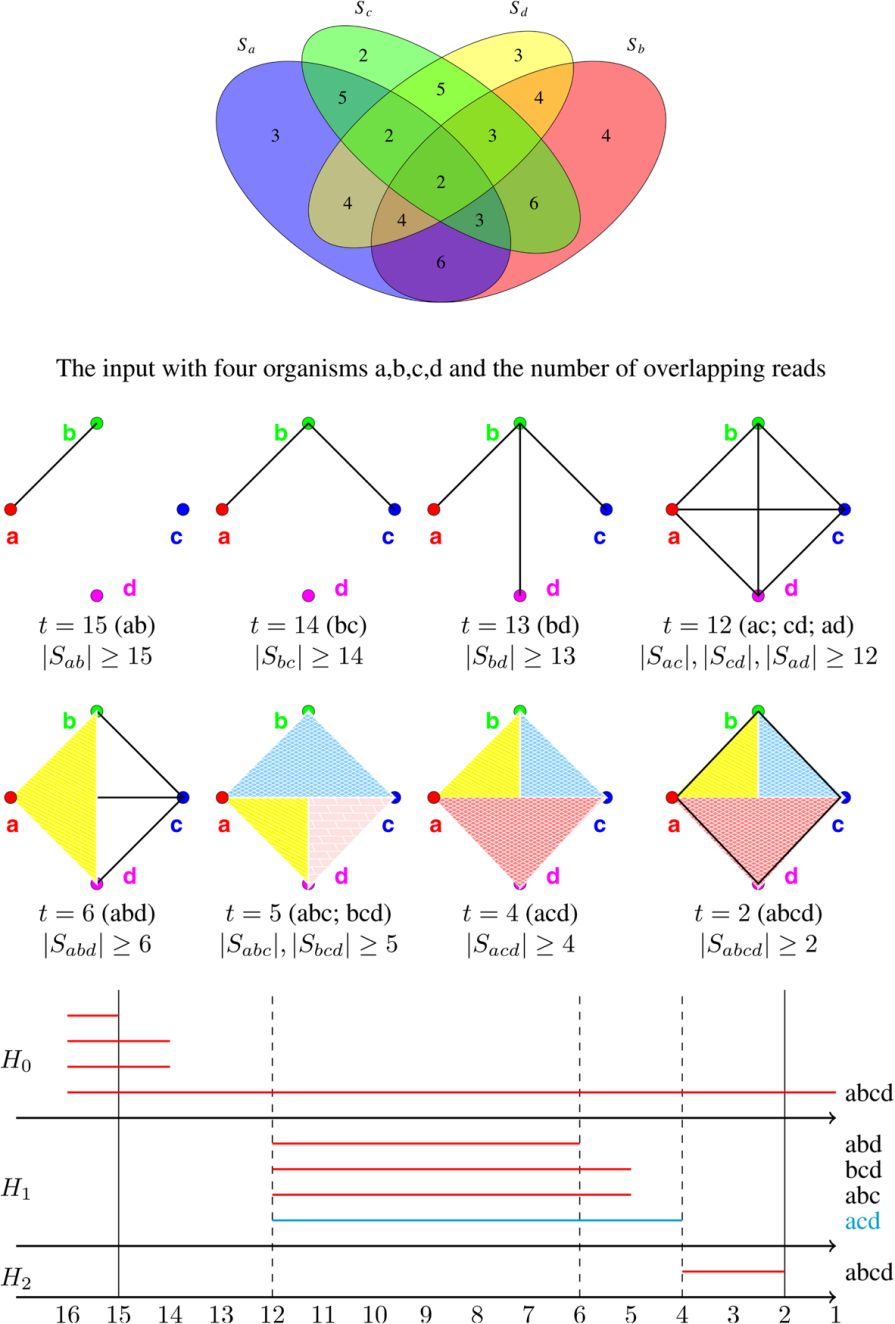
Four organisms and the filtrations on the associated Čech complex with *t*=15 down to *t*=2

A simple example with four organisms is shown in Fig. 4.

## Results

We applied the model on simulated shotgun sequencing reads from a collection of 36 recently published *Salmonella* genomes [15], in an effort to study the applicability of the approach to strain-level detection. We simulated 150 bp paired end reads, 100,000 per each input genome, with dwgsim [16] (with parameters *y*0,*e*0.005,*E*0.005,*d*500,*s*0,*r*0.001,*R*0.15,*X*0.3) from the 36 genomes. The reads were mapped to a database consisting of the same set of genomes using bowtie2 [17] (very-sensitive-local mode, searching for up to 101 hits per read). Read simulation and mapping was performed with the Metagenomics Computation and Analytics Workbench (MCAW) [18].

The mapped reads were processed with custom scripts to prepare the Barycentric depth values and Čech set sizes for each set of organisms. Concordance of paired reads was checked (both reads of a pair mapping to the same organism), a random representative selected if the organism had several hits from the same read, and only the best quality hits per read pair were used (based on sum of edit distances of reads in a pair). In this proof of concept we focused on the set of reads shared among 1 (unique reads), 2, or 3 organisms; the simulated data had 1–3 truly present organisms.

Indicative of the shared genome content between the closely related sequences, between only 2,087 to 92,176 of the 100,000 reads simulated per genome uniquely mapped to that genome after the process described above. Looking only at unique read counts per genome would certainly yield erroneous order of certain strains. For example, we observed a simulated mixture of 3 strains where two false positives (FPs) had more unique reads (2,596 and 2,427) than one of the true positives (TPs) (2,105). In this case, relying only on the number of unique reads would miss the truly present strain and falsely indicate the presence of missing ones. The observed unique reads for false positive organisms can arise from sequencing read errors, effects of the bioinformatics pipeline, and from subtle differences between the reference genomes and the observed genomes.

For analysis, the 36 *Salmonella* genomes were split into 18 non-overlapping sets of 2 strains each, and into 12 sets of 3 strains each. The TDA methodology, including the voting schemes described in Eqs. 6 and 8, was applied to the simulated reads from each set. The resulting voting values *v* for each organism were ordered from large (indicating truly present organisms) to small (indicating potential false positives).

**Fig. 5:**
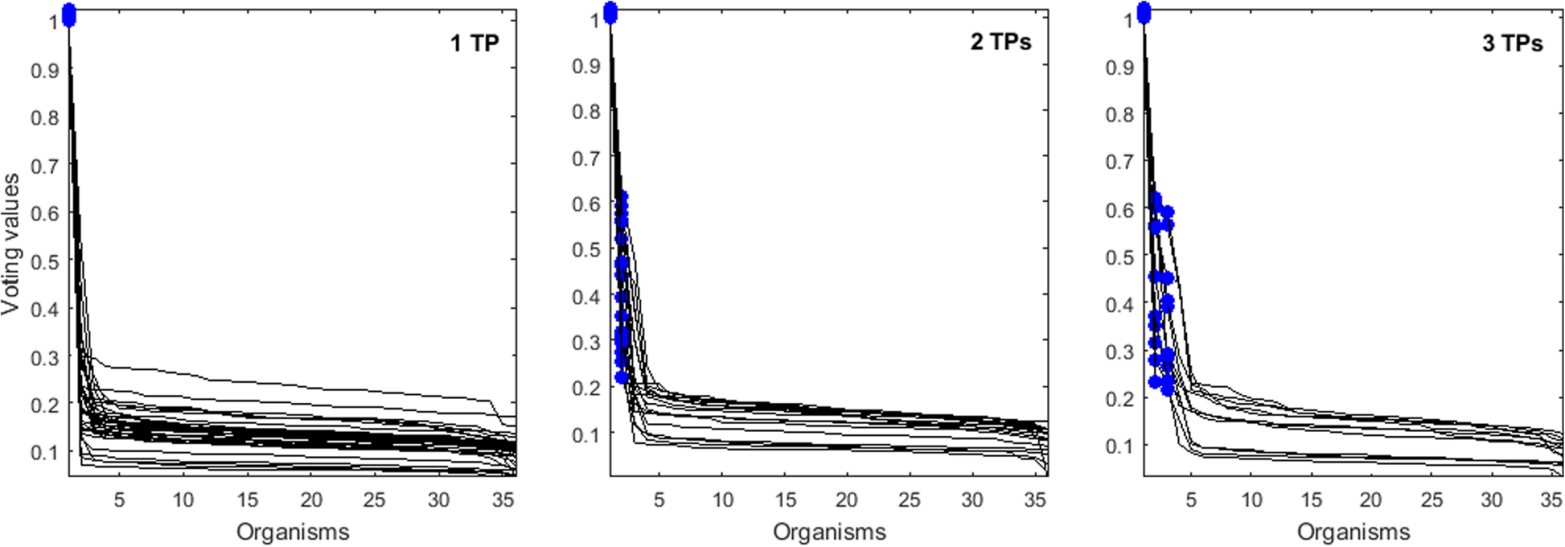
Voting values (vertical axis) according to the Barycentric TDA method. The organisms (horizontal axis) are ordered according to their voting values from Eq. 6. Each line corresponds to a case where simulated reads from 1, 2, or 3 true positive (TP) input organisms were mixed together. The blue filled circles represent the voting values for the TPs

**Fig. 6:**
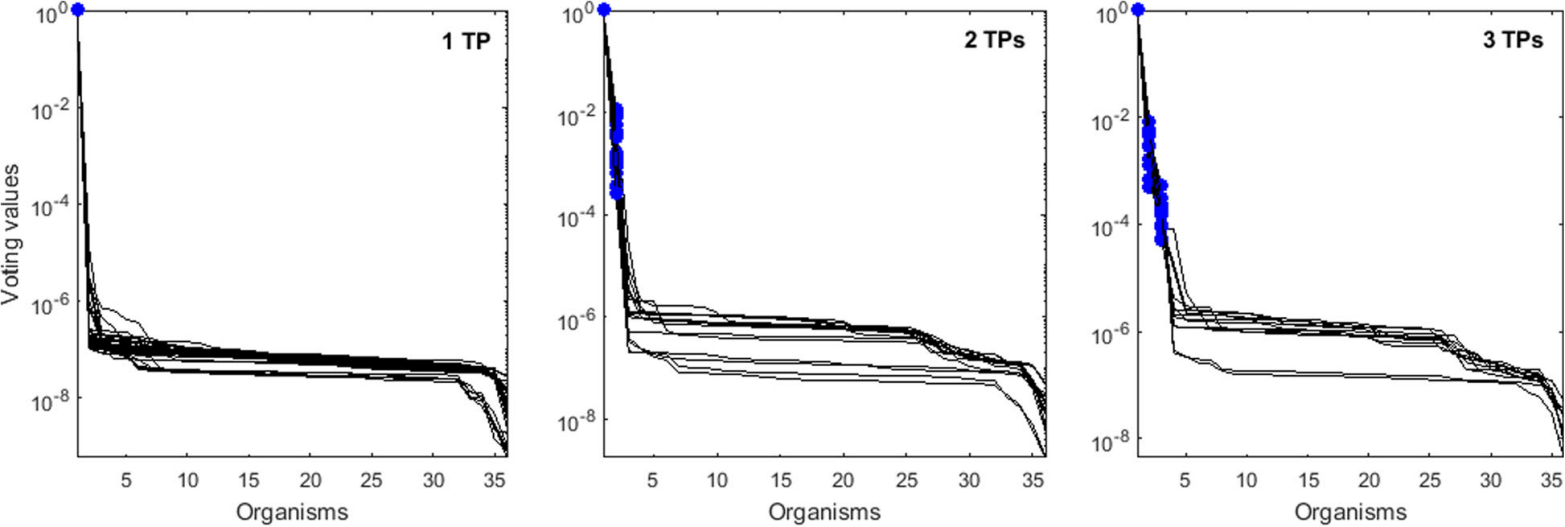
Voting values (vertical axis) according to the Čech TDA method. The organisms (horizontal axis) are ordered according to their voting values from Eq. 8. Each line corresponds to a case where simulated reads from 1, 2, or 3 true positive (TP) input organisms were mixed together. The blue filled circles represent the voting values for the TPs

The voting values for simulated mixtures of 1, 2, or 3 *Salmonella* strains are visualized in Fig. 5 for the Barycentric approach and in Fig. 6 for the Čech approach. Ordering the organisms by voting values perfectly delineates the TPs from FPs with both methods. As a comparison, ordering the organisms by read coverage (computed by bedtools [19]) of the same reads as in the TDA input, the TPs could also be separated from FPs. However, as discussed earlier, using only the uniquely mapping reads would lead to false positives. We demonstrated that the TDA approach solves the problem of enriching the top of the list of voting values for truly present organisms.

## Discussion and conclusions

In this proof of concept study we apply topological data analysis to the problem of separating signal from noise in the analysis of frequently multi-mapping metagenomic sequencing reads. Our approach is based on the construction of a particular subcomplex of a Barycentric subdivision complex, to rank-order the potential organisms and tease out the truly present ones.

The results from applying the approach on simulated genome mixtures show not just separation of signal from noise but also the potential for identifying microbes from metagenome samples, at strain level. We demonstrate the power of the TDA approach even in cases where alternative algorithms that exploit uniquely mapping reads fail. In fact, for the simple test cases the TPs bubble to the top from amongst a reference collection of highly related organisms, indicating promise of success for complicated real-life scenarios. The Čech model that uses less information is equally effective, suggesting that even partial information when augmented with the appropriate structure is quite powerful.

Additionally, the voting value curves show patterns of sharp decrease after the last true positive, suggesting automated calculation of cut-off thresholds. The same methodology could also be used to study higher taxonomic levels, e.g., separating true from false positive genera, families, etc. by modeling the read mappings across taxa.

Our next steps are to scale the implementation, and to apply it in the food safety as well as in the human health contexts. In both applications, a precise strain level assignment is of paramount importance.

## Abbreviations

FP: False positive
ROM: Reads-to-organism mapping
TDA: Topological data analysis
TP: True positive
